# *Meg8*-DMR as the Secondary Regulatory Region Regulates the Expression of MicroRNAs While It Does Not Affect Embryonic Development in Mice

**DOI:** 10.3390/genes14061264

**Published:** 2023-06-14

**Authors:** Liang Zhang, Zhengbin Han, Hongjuan He, Ximeijia Zhang, Mengyan Zhang, Boran Li, Qiong Wu

**Affiliations:** 1 School of Life Science and Technology, Harbin Institute of Technology, Harbin 150001, China; 2 State Key Laboratory of Urban Water Resource and Environment, Harbin Institute of Technology, Harbin 150001, China

**Keywords:** genomic imprinting, differentially methylated region, CRISPR/Cas9, *Meg8*-DMR, *Dlk1-Dio3* domain

## Abstract

*Meg8*-DMR is the first maternal methylated DMR to be discovered in the imprinted *Dlk1-Dio3* domain. The deletion of *Meg8*-DMR enhances the migration and invasion of MLTC-1 depending on the CTCF binding sites. However, the biological function of *Meg8*-DMR during mouse development remains unknown. In this study, a CRISPR/Cas9 system was used to generate 434 bp genomic deletions of *Meg8*-DMR in mice. High-throughput and bioinformatics profiling revealed that *Meg8*-DMR is involved in the regulation of microRNA: when the deletion was inherited from the mother (Mat-KO), the expression of microRNA was unchanged. However, when the deletion occurred from the father (Pat-KO) and homozygous (Homo-KO), the expression was upregulated. Then, differentially expressed microRNAs (DEGs) were identified between WT with Pat-KO, Mat-KO, and Homo-KO, respectively. Subsequently, these DEGs were subjected to the Kyoto Encyclopedia of Genes and Genomes (KEGG) pathway and Gene Ontology (GO) term enrichment analysis to explore the functional roles of these genes. In total, 502, 128, and 165 DEGs were determined. GO analysis showed that these DEGs were mainly enriched in axonogenesis in Pat-KO and Home-KO, while forebrain development was enriched in Mat-KO. Finally, the methylation levels of IG-DMR, *Gtl2*-DMR, and *Meg8*-DMR, and the imprinting status of *Dlk1*, *Gtl2,* and *Rian* were not affected. These findings suggest that *Meg8*-DMR, as a secondary regulatory region, could regulate the expression of microRNAs while not affecting the normal embryonic development of mice.

## 1. Introduction

Genomic imprinting is an epigenetic mechanism in mammals that leads to the functional inequivalence of the parental genome [1]. The imprinted genes are regulated by differentially methylated regions (DMRs), which are dependent on the parent of origin. Imprinted genes are mainly characterized by a single allelic expression [2,3,4]. The differential methylation patterns originate during spermatogenesis or after fertilization [5]. A loss of imprinting can lead to the silencing or biallelic expression of genes, which leads to various embryonic developmental defects and postnatal diseases. The imprinted *Dlk1*-*Dio3* domain that is located on the distal arm of mouse chromosome 12 and human chromosome 14 contains three paternally expressed protein-coding genes (*Dlk1*, *Rtl1*, and *Dio3*), four maternally expressed long non-coding RNA genes (*Gtl2*, *anti*-*Rtl1*, *Rian*, and *Mirg*) [6,7,8,9,10,11,12,13], and a large number of microRNAs and snoRNAs [14,15]. The Dlk1-Dio3 imprinting region in mammals has been reported as highly conserved in humans and mice, including chromosome localization, sequence characteristics, and gene imprinting status. In addition, imprinted genes within the Dlk1-Dio3 domain have also been reported to be imprinted conserved in other mammals, such as sheep, cattle, and pigs [16,17]. These imprinted genes have been shown to have important functions in controlling prenatal development and postnatal differentiation.

The *Dlk1*-*Dio3* imprinted domain contains three paternally methylated DMRs (IG-DMR, *Gtl2*-DMR, *Dlk1*-DMR) and a functionally unknown maternally methylated differentially methylated region, *Meg8*-DMR [18,19]. The *Dlk1*-*Dio3* domain is mainly regulated by IG-DMR, which is methylated during spermatogenesis [20]. In mice, when the IG-DMR knockout occurs on paternal alleles, the offspring are born with normal phenotype and gene expression. However, when it occurs on maternal alleles, this results in perinatal lethality. The expression of *Dlk1*, *Rtl1*, and *Dio3* all become biallelic, while maternally expressed genes are suppressed [21,22]. The maternally expressed genes of the *Dlk1*-*DIo3* domain are secondarily regulated by *Gtl2*-DMR. In mice, when the *Gtl2*-DMR knockout occurs on a maternal allele, the offspring are born with a normal phenotype. However, all the offspring die within four weeks, mainly due to severe hypoplastic pulmonary alveoli and hepatocellular necrosis. When the deletion occurs on the paternal allele, the embryos show severe developmental disabilities and high lethality in the perinatal period [23]. The *Meg8*-DMR located inside the second intron of *Rian* (*Rian* and *Meg8* are alternative names given to the same transcript) is the first maternally methylated, somatically derived, differentially methylated region and acquired differential methylation prior to E7.5 in the domain. In addition, the CTCF binds to the *Meg8*-DMR non-allele, specifically in vivo. Deletion of the *Meg8*-DMR in the mouse MLTC-1 cell line enhanced cell migration and invasion [24]. A previous study found that *Meg8*-DMR shows hypermethylation in TS14 patients, while hypomethylation occurred in KOS14 patients [25]. The methylation levels in the brain of Temple syndrome patients are quite interesting. The methylation levels are very low in fetal and 4-month-old patient brains [26]. However, for patients aged from 44 to 79 years, methylation levels are 26–30%. Thus, it seems that the methylation level in the brain increases with age [27]. To the best of our knowledge, *Meg8*-DMR will be the first reported imprinting site to obtain methylation in a tissue-specific manner with age. In addition, the methylation level may be associated with the development of intelligence in patients with Temple syndrome. However, little is known about the role of *Meg8*-DMR in mice.

To investigate the biological function of *Meg8*-DMR, the CRISPR/Cas9 system was used to generate genomic deletions in mice with paternal deletion, maternal deletion, and the parental double-deletion of *Meg8*-DMR. The weight, mode of inheritance, morphology, and methylation levels are not changed. The expression level of multiple microRNAs is exchanged. High-throughput analysis showed that differentially expressed genes are enriched in axonogenesis. These results imply that somatic-cell-derived *Meg8*-DMR could regulate the expression of microRNAs while not affecting the normal embryonic development of mice.

## 2. Materials and Methods

### 2.1. Generation of Meg8-DMR Mice

A sgRNA DNA temple was generated using PCR amplification with the following primers: sgRNA F-5′-TTAAGATTTGTGCCAACTCA-3′ and sgRNA R-5′-CAGGAGCCCAGGAAACCCAA-3′. PCR products were purified using Qiaquick (#28014, Qiagen, Frankfurt, Hessian, Germany) columns, and approximately 100 ng of DNA was used as a template for the in vitro transcription reaction of T7 (#AM1344, Invitrogen, Carlsbad, CA, USA). In vitro transcription and Cas9/sgRNA co-injection were performed as described previously. DBA and C57BL6/J mice were mated to produce the hybrid strain DBAB6F1. The *Meg8*-DMR knockout mice lines were maintained by mating heterozygous mice with ICR mice. The Pat-KO, Mat-KO, and WT mice were generated by interbreeding with WT and heterozygous mutants. The Homo-KO mutants were obtained by mating male and female heterozygous mutants. Genotype identification was carried out using PCR amplification with the following primers: *Meg8*-F-5′-CAACAGGATAACTCTGGCAACTTCC-3′and *Meg8*-R-5′-CTAACCCCTTATGACTCTCCATCGT-3′.

### 2.2. RNA Extraction and qRT-PCR

Total RNA was extracted from the tissue using RNAiso Plus (TaKaRa, Dalian, China). The cDNA was synthesized with reverse transcription using the PrimeScriptTM RT kit (Takara). qRT-PCR was performed on the ABI 7500 real-time fluorescent quantitative PCR system using FastStart Universal SYBR Green Master (Roche, Basel, Switzerland). The primers used for the analysis are shown in Table S1. MicroRNA reverse transcription reactions were performed with the TaqMan miRNA Reverse Transcription Kit (#4366596, Applied Biosystems, Carlsbad, CA, USA) and miRNA-specific reverse transcription primers (#4366596, Applied Biosystems, USA), and quantitative PCR was performed with TaqMan 2× Universal PCR Master Mix (#4369016, Applied Biosystems, USA) and miRNA-specific quantitative PCR primers (#4409156, Applied Biosystems, USA).

### 2.3. RNA-Seq Analysis

Five separated total RNAs were extracted from embryos and mixed together. RNA electrophoresis was used for quality detection, and Nanodrop was used for purity analysis. The mixed RNAs were provided to a private Bio Company for transcriptome sequencing. DEGs were defined as the level of gene expression in mutant mice relative to expression in WT mice, and absolute fold change >1 and *p*-value < 0.05 were used to identify DEGs. For DEGs, log2 fold change (FC) was defined as upregulated DEGs if it was positive and downregulated DEGs if it was negative. David Bioinformatics Resources (v6.8, Frederick, MD, USA) was used to annotate the GO functions of different genes.

### 2.4. DNA Methylation Analysis

DNA was isolated from the embryo of WT, Pat-KO, Mat-KO, and Homo-KO mutants. Sequencing was performed as described previously. The primers used were as follows: IG-DMR, forward 5′-GTATTGTAATATAGGTTAGGTG-3′, reverse 5′-CTACATAATACCATATAAACATATCTC-3′. *Gtl2*-DMR, forward 5′-AAATTCTGCAAGGAAAAGAATCCTCAGG-3′, reverse 5′-TTCAGAATTGCTGGTCAACATGAACCTC-3′. *Meg8*-DMR, forward 5′-AGTTGGGTTATTTAAGATTTGTGTT-3′, reverse 5′-AATTTCCTAAACTCCTAACCACTTC-3′.

### 2.5. Allelic Analysis of Gene Expression

According to the analysis of SNP sites in the imprinting region on the bioinformatics website (http://www.informatics.jax.org/strains_SNPs.shtml, accessed on 5 June 2021), SNP sites of *Gtl2* exist between the ICR mouse strain and C57BL/6J mouse strain, and SNP sites of *Dlk1* and *Rian* exist between the ICR mouse strain and DBA/2J mouse strain. Using the DNA of each pure line of mice, the correctness of SNP sites was verified. Two different hybrid lines were crossed. The pregnant mice were dissected in E12.5 and E15.5, and two groups of hybrid lines were obtained: BIF1 and IBFI and IDF1 and DIF1, respectively. The total RNA of the E12.5 embryo and E15.5 tissues were extracted, and the cDNA was obtained with reverse transcription, which was used as an experimental sample to establish the crossed cDNA library. At the same time, the DNA of the parents of each hybrid line and pure line was extracted. cDNA primers and DNA primers containing SNP sites were designed in the detected imprinted gene sequences. DNA samples of parents and hybrids were PCR and product recovery sequencing, and SNP sites of bioinformatics analysis were verified. Then, cDNA bit templates of hybrids were used for PCR product recovery sequencing.

## 3. Results

### 3.1. CRISPR/Cas9-Mediated Meg8-DMR Deletion in Mice

**Figure 1:**
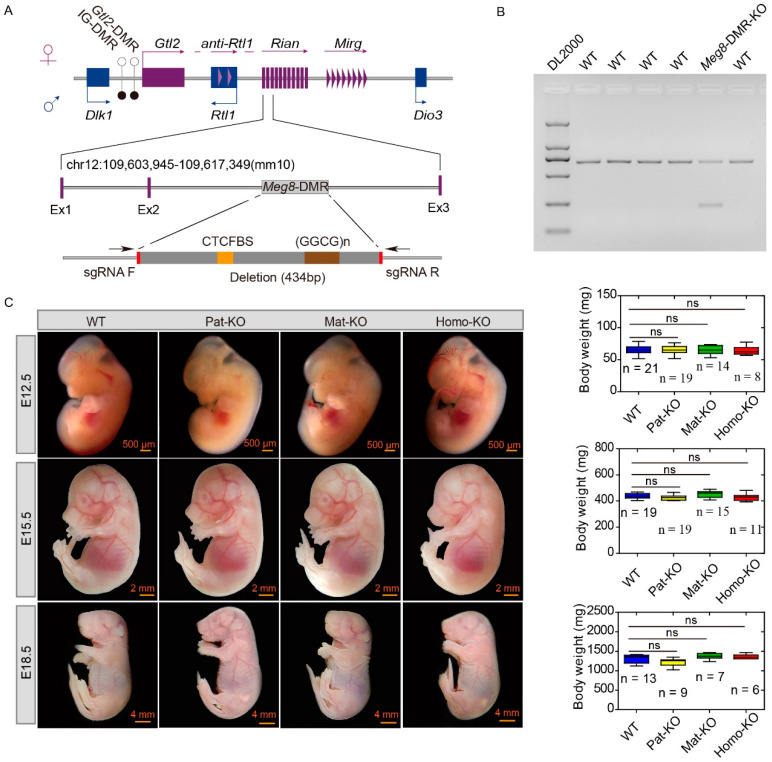
CRISPR/Cas9-mediated deletion of *Meg8*-DMR and the growth of the mutants. (**A**) Regional physical map of the *Dlk1*-*Dio3* domain and deletion locus of *Meg8*-DMR on mouse chromosome 12. The positions of imprinted genes are indicated by squares, vertical bars (snoRNAs), or triangles (microRNA). Maternally expressed genes are indicated by purple, paternally expressed genes are indicated by blue, CTCFBS is indicated by yellow and (GGCG)n is indicated by brown. The sgRNA primers are indicated by red. The direction of sgRNA primers are indicated by the arrows. IG-DMR and *Gtl2*-DMR are indicated by circles (filled circles, hypermethylation; open circles, hypomethylation). (**B**) Demonstration of 434bp deletion in the *Meg8*-DMR in founder animals. One of the six founders showed a 434bp deletion in the *Meg8*-DMR. (**C**) Gross phenotype of E12.5, E15.5, and E18.5 *Meg8*-DMR embryos. Comparison of WT with Pat-KO, Mat-KO, and Homo-KO mutants.

To investigate the functional role of the *Meg8*-DMR in mice, the CRISPR/Cas9 system was used to knockout the *Meg8*-DMR. The CRISPR/Cas9 system with two sgRNAs (sgRNA F and sgRNA R) was used to induce a 434 bp deletion in the *Meg8*-DMR. Cas9 mRNA and sgRNAs were transcribed in vitro. Then, they were injected together into blastocyst-stage embryos (Figure 1A). In total, six pups were successfully obtained from 83 transferred embryos. The primers *Meg8*-F and *Meg8*-R were used to validate the deletion of *Meg8*-DMR. PCR products indicated that one of the six founders harbored the deletion (Figure 1B). The PCR product was cloned into TA cloning vectors and further confirmed by sequencing. Sequencing of this pup showed that 434 bp between the two targeted sites was successfully knocked out. To investigate whether deletions could be stably inherited by pups, the founder was mated with wild-type ICRs. The genotypic identification of litters from F1 animals showed that *Meg8*-DMR deletion can be stably inherited by their offspring.

### 3.2. The Mode of Inheritance of Meg8-DMR Mutants

To investigate the epigenetic mode of *Meg8*-DMR knockout mice in the embryos, we generated four genotypes of E12.5 embryos, WT, Homo-KO, Pat-KO, or Mat-KO by crossing male *Meg8*-DMR heterozygous with female heterozygous mutants. However, it was impossible to distinguish whether the deletion was from the paternal or maternal allele. By crossing male wild-type mice with female heterozygotes mice, we generated Mat-KO embryos, and by crossing male heterozygotes with female wild-type mice, we generated Pat-KO embryos. Compared with the wild-type, Pat-KO, Mat-KO, and Homo-KO mutant embryos had normal physical characteristics in terms of body weight and length (Figure 1C). In order to eliminate the interference caused by the cohabitation scheme, we tried to combine the mating scheme. The results were consistent: the number of pups and proportion of each genotype were close to the Mendelian inheritance proportion in the *Meg8*-DMR mutants (Table 1). To investigate whether *Meg8*-DMR knockout results in perinatal lethality, E15.5 embryos and E18.5 embryos were generated in the same crossing. The mode of inheritance and phenotype were the same as the E12.5 mutant embryos. Furthermore, all the mutants survived at the embryonic stage and developed into normal adults with reproductive abilities.

### 3.3. Analysis of DEGs in WT and CRISPR/Cas9-Mediated Meg8-DMR Knockout Mice

**Figure 2:**
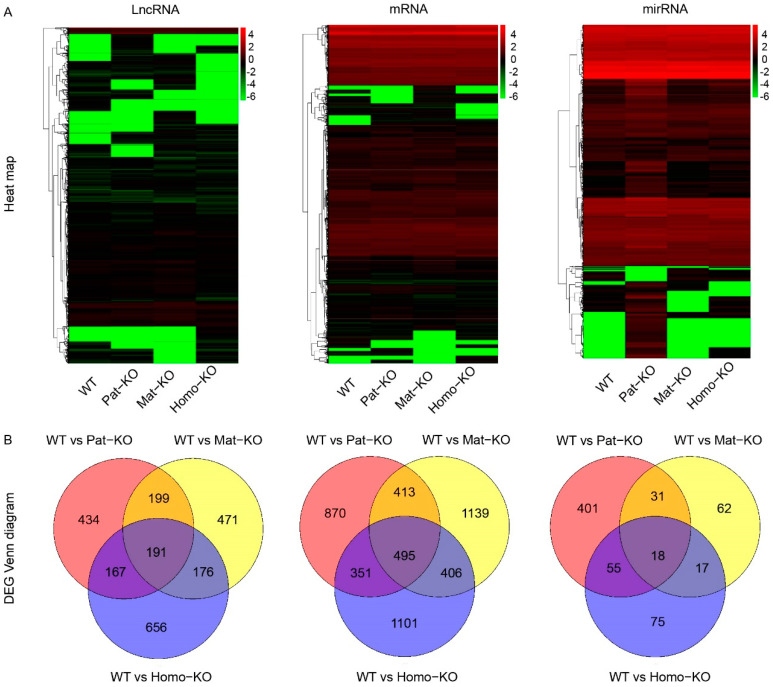
Analysis of differentially expressed genes (DEGs) in WT and CRISPR/Cas9-mediated *Meg8*-DMR knockout mice. (**A**) Heat map of LncRNA, mRNA, and miRNA DEGs in WT and *Meg8*-DMR knockout mice. From left to right: lncRNA, mRNA, and microRNA; The horizontal coordinate represents the sample name and the clustering result of the sample, and the vertical coordinate represents the differential gene and the clustering result of the gene. Different columns in the diagram represent different samples, and different rows represent different genes. The color represents the level of gene expression in the sample log10 (gene + 0.000001). (**B**) Venn diagram of DEGs in Pat-KO (red), Mat-KO (yellow), and Homo-KO (blue) mutants compared to the wild-type. From left to right: lncRNA, mRNA, and microRNA.

To further investigate the molecular mechanism underlying *Meg8*-DMR regulation, RNA-seq was performed to analyze E12.5 embryos. We performed a differentially expressed genes’ (DEGs’) heat map analysis of lncRNA, mRNAs, and microRNAs in total embryonic RNA from each of the four genotypes (Figure 2A). The results showed that microRNA expression profiles were significantly different in Pat-KO, Mat-KO, and Homo-KO compared with WT. To our surprise, microRNAs, following *Meg8*-DMR knockout, had the greatest impact on Pat-KO, followed by Homo-KO and Mat-KO. This result was consistent with the pattern of imprinting regulation. A subsequent Venn diagram analysis of microRNA expression profiles was conducted; the threshold was ∣log2 fold change (FC)∣ > 1 and adjusted *p* value < 0.05. The results showed 505, 128, and 165 DEGs for Pat-KO, Mat-KO, and Homo-KO, respectively, compared with WT (Figure 2B). Overall, *Meg8*-DMR knockout had broad effects on microRNA expression profiles.

### 3.4. Detection of miRNA DEGs in WT and Meg8-DMR Knockout Mice

**Figure 3:**
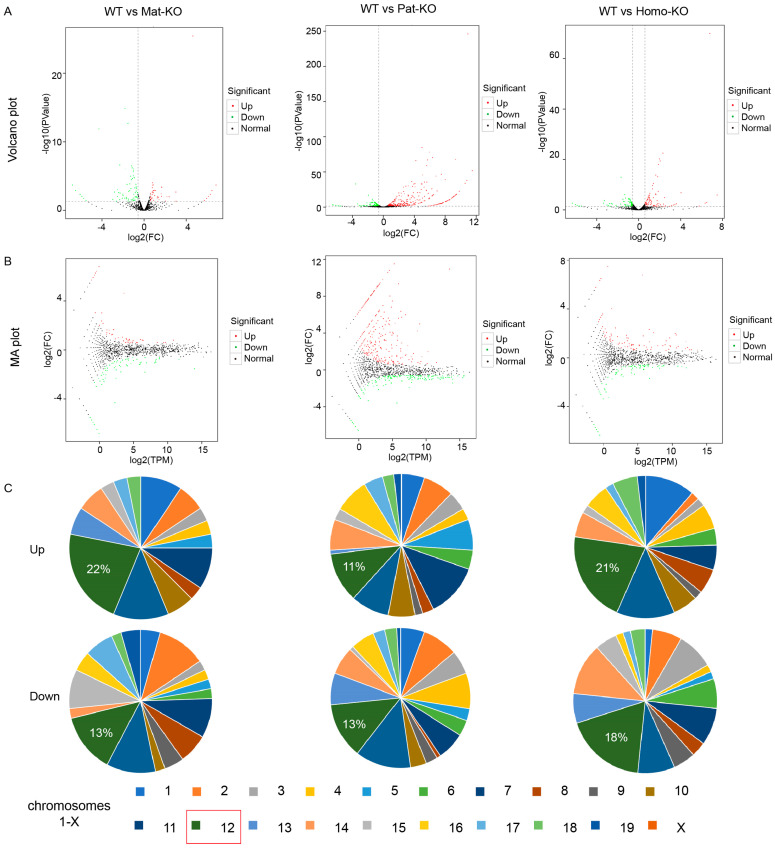
Detection of miRNA DEGs in WT and *Meg8*-DMR knockout mice. (**A**) Volcano plot corresponding to the WT, Pat-KO, Mat-KO, and Homo-KO. From left to right: WT vs. Pat-KO, WT vs. Mat-KO, and WT vs. Homo-KO. (**B**) Meandiff plot for the WT compared with Pat-KO, Mat-KO, and Homo-KO. (**C**) Distribution of upregulated and downregulated miRNAs in Pat-KO, Mat-KO, and Homo-KO on chromosomes.

To learn more about the involvement of *Meg8*-DMR in microRNA regulation, we obtained the DEGs of Pat-KO, Mat-KO, and Homo-KO and compared them with WT. The DEGs are presented in a volcano plot (Figure 3A), and the chromosome distribution of the DEGs are presented in the pie charts (Figure 3B). The red dots represented upregulated DEGs, and the green dots represented downregulated DEGs. In total, 152 upregulated and 353 downregulated DEGs were identified in WT vs. Pat-KO, 77 upregulated and 51 downregulated DEGs were identified in WT vs. Mat-KO, and 79 upregulated and 86 downregulated DEGs were identified in WT vs. Homo-KO. In comparison to the WT, the majority of upregulated and downregulated DEGs were found on chromosome 12, where the *Meg8*-DMR was located.

### 3.5. GO and KEGG Pathway Enrichment of DEGs

**Figure 4:**
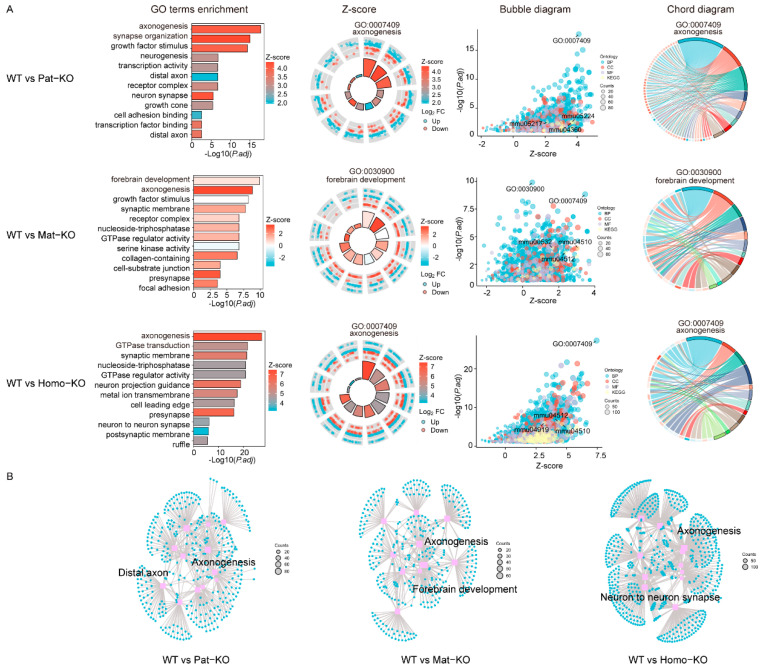
Go and KEGG pathway enrichment of DEGs. (**A**) GO terms and KEGG pathway enrichment results. From left to right: the top 12 GO enrichment results and Z-score results; bubble diagram and relationships between DEGs and the KEGG pathway. (**B**) Network diagram and relationships between DEGs and the top 12 GO enrichment results.

To further understand the effects of *Meg8*-DMR at the embryonic stage, we compared Pat-KO, Mat-KO, and Homo-KO mutants with WT using GO and KEGG pathway enrichment analysis. We used the top 12 GO terms for comparative analysis. The results of microRNA-associated DEGs analysis in Pat-KO and Homo-KO suggest that the biological processes of these genes were mainly involved in axonogenesis and forebrain development in Mat-KO (Figure 4A). The network diagram and relationships between DEG and the top 12 GO enrichment results showed that axonogenesis was located at the central node of the network (Figure 4B). Taken together, the GO and KEGG pathway enrichment analysis of DEGs in mutations revealed that *Meg8*-DMR played a role in the axonogenesis and forebrain development of mice.

### 3.6. Expression of the Imprinted Genes in the Dlk1-Dio3 Domain

**Figure 5:**
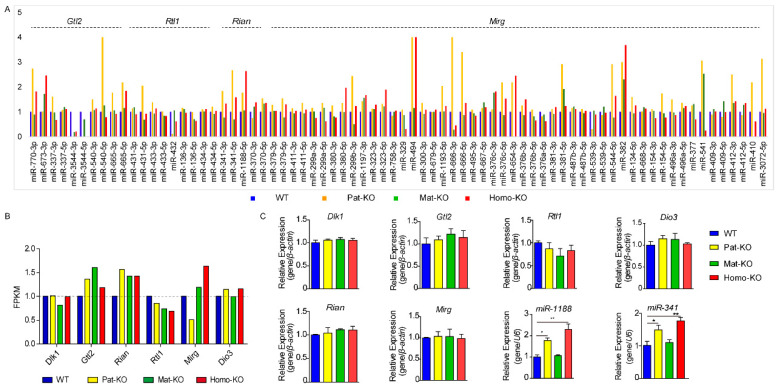
Expression of the imprinted genes in the *Dlk1*-*Dio3* domain. (**A**) TPM analyses of the microRNA expression in the *Dlk1*-*Dio3* domain. (**B**) FPKM analyses of the lncRNA and mRNA expression in the *Dlk1*-*Dio3* domain. (**C**) The expression level of the six imprinted genes *Dlk1*, *Gtl2*, *Rtl1*, *Rian*, *Mirg*, *Dio3*, and two microRNA mir-1188; mir-341 was measured at E12.5. Error bars, mean ± SD. *n* = 4. *p* values were calculated using two-way ANOVA. ** *p* < 0.01, * *p* < 0.05.

The *Dlk1*-*Dio3* domain contains a number of microRNAs. We also analyzed the effect on microRNA on the cluster. RNA-seq showed that *Meg8*-DMR knockout resulted in altered expression of the microRNA. To our surprise, microRNA expression was upregulated in Pat-KO and Homo-KO mutants, whereas microRNA in Mat-KO mutants showed little change compared with WT (Figure 5A). The maternally expressed *Gtl2* and *Rian* were upregulated and paternally expressed; *Rtl1* was downregulated. The expression of *Dlk1*, *Mirg*, and *Dio3* did not differ from that of WT (Figure 5B). QRT-PCR analysis showed that the expression of *Gtl2* was increased by 8% in Pat-KO, 14% in Mat-KO, and 12.4% in Homo-KO compared to WT. The expression of *Rian* was increased by 3.7% in Pat-KO, 10% in Mat-KO, and 9.8% in Homo-KO. The expression of *Rtl1* was decreased by 8.6% in Pat-KO, 7.1% in Mat-KO, and 8.3% in Homo-KO. The expression of *Dlk1*, *Mirg*, and *Dio3* was not altered. QRT-PCR analysis of miR-1188 and miR-341 showed that the expression of miR-1188 was increased by 77% in Pat-KO, 6% in Mat-KO, and 120% in Homo-KO compared with WT. The expression of miR-341 was increased by 68% in Pat-KO, 5% in Mat-KO, and 90% in Homo-KO (Figure 5C), the primer sequences were shown in Table S1. In summary, the paternal expression gene *Rtl1* was downregulated, and maternal expression genes *Gtl2* and *Rian* were upregulated in the *Dlk1*-*Dio3* domain; the expression of microRNA was upregulated in Pat-KO and Homo-KO mutations; little changed in Mat-KO.

### 3.7. Methylation Status and Allelic Analysis of Gene Expression in the Meg8-DMR Mutants

**Figure 6:**
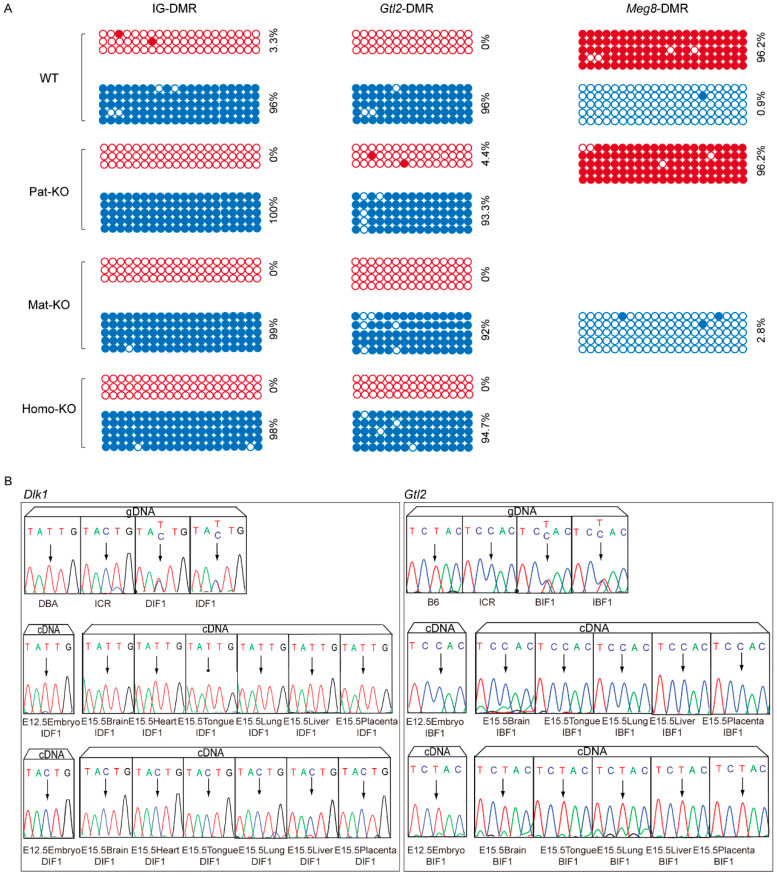
Methylation status and allelic analysis of gene expression in the *Meg8*-DMR mutants. (**A**) IG-DMR, *Gtl2*-DMR, and *Meg8*-DMR methylation status in the *Meg8*-DMR mutants. Maternal allele and paternal allele are represented by red and blue circles, respectively. Methylated and unmethylated CpG sites are represented by filled and open circles. (**B**) Sequence analysis of the PCR products of *Dlk1*, *Gtl2*, and *Rian*. The samples were obtained from genomic DNA (*Dlk1* was obtained from DBA/2J and ICR; *Gtl2* was obtained from C57BL/6J and ICR) and cDNA. The polymorphic bases in the sequences are arrowed.

The *Dlk1*-*Dio3* domain was mainly regulated by IG-DMR and *Gtl2*-DMR. We examined whether *Meg8*-DMR knockout changed the methylation status. We found that the paternal methylation levels of IG-DMR and maternal methylation levels of *Gtl2*-DMR were not changed. The methylation status of Pat-KO, Mat-KO, and Homo-KO mutants was consistent with that of WT. Then, we investigated the methylation level of *Meg8*-DMR in WT, Pat-Ko, and Homo-KO mutants. The results showed the hypermethylation level of the maternal allele in Pat-KO mutants, while the unmethylation level of paternal alleles was shown in Mat-KO mutants (Figure 6A). These results indicated that the deletion of Meg8-DMR does not change the methylation status of IG-DMR, *Gtl2*-DMR, and *Meg8*-DMR.

To further understand the expression status of imprinting genes in *Meg8*-DMR knockout mutants, we analyzed the imprinting status of *Dlk1* and *Gtl2* in transgenic mice. The imprinting patterns in E12.5.5 whole embryo and E15.5 brain, tongue, lung, liver, and placenta tissues were detected. The SNP sites of *Dlk1* (T in DBA/2J strain, C in ICR strain) and Gtl2 (T in C57BL/6, C in ICR strain) marked the parents of hybrid mice, respectively, and the DNA of embryos and tissues of IDF1 and DIF1 were detected. The peak pattern at the selected SNP sites showed double peaks. The expression of paternally expressed gene *Dlk1* was only expressed by the paternal allele, while the expression of the maternally expressed gene *Gtl2* was only expressed by the maternal allele (Figure 6B). These results indicated that the imprinting status of *Dlk1* and *Gtl2* in the whole embryo and tissues was not changed.

## 4. Discussion

*Meg8*-DMR, the first discovered maternally methylated DMRs within the *Dlk1*-*Dio3* imprinting domain, has not been well-studied. In this study, we generated *Meg8*-DMR knockout mouse models using the CRISPR/Cas9 system and obtained WT, Pat-KO, Mat-KO, and Homo-KO mutants using genotypic hybridization. The characterization of these mice suggested that the deletion of *Meg8*-DMR caused the expression of microRNA to be upregulated in Pat-KO and Homo-KO mutations, while little changed in Mat-KO. However, the effect is not sufficient to have a significant impact on morphological characteristics or embryonic development. This provides new insight into the role of *Meg8*-DMR in embryonic development.

The current understanding of DMRs within the *Dlk1*-*Dio3* domain was mainly based on IG-DMR and *Gtl2*-DMR. However, the genetic pattern, characterization, and molecular performance of *Meg8*-DMR at the embryonic stages were significantly different from those of IG-DMR and *Gtl2*-DMR. *Meg8*-DMR did not affect the development of mouse embryos, as Meg8-DMR was a somatic cell-derived DMR. The expression of lncRNA, mRNA, microRNA, and snoRNAs in the imprinted domain changed when IG-DMR or *Gtl2*-DMR were knocked out. However, the expression of microRNAs changed most significantly after *Meg8*-DMR deletion, indicating that the regulatory ability of *Meg8*-DMR was the weakest. In addition, the methylation status of DMRs in *Dlk1*-*Dio3* was not altered; it seems that the transcriptional regulation altered the expression of microRNAs via the *cis* and *trans* effects [28].

In this study, the expression of *Gtl2*, *Rtl1*, and *Rian* changed in all *Meg8*-DMR mutants, although the dosage was less than 10%. This suggested that both chromosomes derived from this parent of *Meg8*-DMR may have biological functions. This bears some resemblance to the unique phenotype of *Gtl2* knockout mice. A previous study found that the effect on mice was lethal during the embryonic period when *Gtl2* deletion occurred in Pat-KO. When *Gtl2* deletion occurred in Mat-KO, all mice died within 4 weeks of birth. It is likely that this regulatory relationship requires the process of transcription or the transcript itself. We hypothesized that the changes induced by *Meg8*-DMR knockout in the embryo would continue to accumulate until the mice were born or grew into adult mice.

Our previous experiments showed that the same gene expression pattern could be observed in MLTC-1 cells by knocking out only the CTCF binding site or all the *Meg8*-DMR [24]. In particular, the paternally expressed genes Dlk1 and Rtl1 were significantly downregulated. The invasion and migration abilities of MLTC-1 cells were enhanced. However, the changes were only insignificant when GGCG repeats were knocked out. This indicates that the GGCG repeats are required for the function of *Meg8*-DMR. In MLTC-1, we knocked out 644bp, including the GGCG repeats, but only knocked out 434bp when the knockout occurred in mice. We suspect that the deletion of the 210bp nucleotide sequence may be the main cause of the phenotypic difference.

In this study, GO and KEGG pathway enrichment showed that axonogenesis and forebrain development were enhanced, although this phenotype was not present in the embryonic development period. Axonogenesis and forebrain development led to a change in neural connections and early hippocampal development. This is essential for neural conduction, spatial learning, and memory formation in later cognitive functioning [29,30]. High-throughput sequencing analysis revealed that genes associated with hippocampal development, such as NFAT and *Ndufb1*, were upregulated. Moreover, the intraventricular injection of adenovirus and overexpression of *Rian* can improve sevoflurane-induced neuronal injury and apoptosis, and the activity of the hippocampal neurons is enhanced in this process. Therefore, *Rian* has a neuroprotective effect on cognitive dysfunction [31]. In this study, the expression of Rian tended to be upregulated at the embryonic stage. This suggests that *Meg8*-DMR may be associated with the development of intelligence in adult mice. This study identified *Meg8*-DMR as a potential target for improving cognitive performance in adult mice.

## Figures and Tables

**Table 1:** Number of pups per litter in the *Meg8*-DMR mutants.

Mating Pattern	No. of Litters		No. of Pups/Total (%)			
		Total	WT	Pat-KO	Mat-KO	Homo-KO
WT (♀) × WT (♂)	3	33	33 ± 0.67 (100)	—	—	—
WT (♀) × Pat-KO (♂)	4	36	18 ± 0.75 (50)	18 ± 1.0 (50)	—	—
WT (♀) × Mat-KO (♂)	3	30	14 ± 0.83 (46.7)	16 ± 0.63 (53.3)	—	—
WT (♀) × Homo-KO (♂)	3	39	—	39 ± 0.66 (100)	—	—
Homo (♀) × WT (♂)	3	37	—	—	37 ± 1.06 (100)	—
Homo (♀) × Pat-KO (♂)	3	34	—	—	15 ± 1.3 (44.1)	19 ± 0.76 (55.9)
Homo (♀) × Mat-KO (♂)	3	30	—	—	14 ± 0.5 (46.7)	16 ± 1.1 (53.3)
Pat-KO (♀) × Pat-KO (♂)	3	24	5 ± 0.5 (20.8)	13 ± 0.66 (54.2)		6 ± 0.66 (25)
Pat-KO (♀) × Mat-KO (♂)	5	64	18 ± 1.12 (28.1)	30 ± 1.2 (46.9)		16 ± 1.04 (25)
Pat-KO (♀) × Homo-KO (♂)	3	36	—	17 ± 0.46 (47.2)	—	19 ± 0.9 (52.8)
Pat-KO (♀) × WT (♂)	5	51	28 ± 1.2 (54.9)	—	23 ± 1.5 (45.1)	—
Mat-KO (♀) × Pat-KO (♂)	4	56	16 ± 1.0 (28.6)	30 ± 1.0 (53.5)		10 ± 1.0 (17.9)
Mat-KO (♀) × Mat-KO (♂)	4	51	12 ± 2.0 (23.5)	28 ± 1.5 (54.9)		11 ± 0.9 (21.6)
Mat-KO (♀) × Homo-KO (♂)	3	39	—	21 ± 1.3 (53.8)	—	18 ± 1.3 (46.2)
Mat-KO (♀) × WT (♂)	3	27	15 ± 0.67 (55.5)	—	12 ± 1.3 (44.5)	—

## Data Availability

RNA-seq sequencing data have been deposited in GEO under accession number.

## Supplementary Materials

The following supporting information can be downloaded at: https://www.mdpi.com/article/10.3390/genes14061264/s1. Table S1. Primer sequences. References [6,19] are cited in the supplementary materials.
