# Supplementary material for: Meg8-DMR as the Secondary Regulatory Region Regulates the Expression of MicroRNAs While It Does Not Affect Embryonic Development in Mice
Source: Genes (Basel). 2023 Jun 14;14(6):1264. doi: 10.3390/genes14061264 (PMC10298023; doi:10.3390/genes14061264)
Supplement: Supplementary file 1 [file genes-14-01264-s001.zip › genes-2355440-supplementary.pdf]

genes

MDPI

Article

Meg8-DMR as the Secondary Regulatory Region Regulates the Expression of MicroRNAs While It Does Not Affect Embryonic Development in Mice

Liang Zhang [1], Zhengbin Han [1], Hongjuan He [1], Ximeijia Zhang [1], Mengyan Zhang [1], Boran Li [1] and Qiong Wu [1,2,*]

[1] School of Life Science and Technology, Harbin Institute of Technology, Harbin 150001, China
[2] State Key Laboratory of Urban Water Resource and Environment, Harbin Institute of Technology, Harbin 150001, China
* Correspondence: kigo@hit.edu.cn; Tel./Fax: +86-0451-86416944

Supplementary Materials

Table S1. Primer Sequences.

Primer Name	Primer Sequence (5′-3′)
Dlk1-F	ACGGGAAATTCTGCGAAATA
Dlk1-R	CTTTCCAGAGAACCCAGGTG
Gtl2-F	CGAGGACTTCACGCACAAC
Gtl2-R	TTACAGTTGGAGGGTCCTGG
Rtl1-F	TCCAAGGAGCATTCGACGTACCAGTGTGACT TACC
Rtl1-R	AGAGGTACCGGATCCGACTCGAGTCGACAT CG
Rian-F	CTGTTGTGCCCTCCCTGGATG
Rian-R	CCAGCTAGGCTGTGTAAATCATC
Mirg-F	GTTGTCTGTGATGAGTTCGC
Mirg-R	GTTCTTGAACATCGCTCC
Dio3-F	CACGGCCTTCATGCTCTGG
Dio3-R	CGGTTGTCGTCTGATACGCA
β-actin-F	TACCACAGGCATTGTGTAGGACT
β-actin-R	TTGATGTCACGCACGATTTCCCT

Supplementary References

1. Rocha, S.T.D.; Edwards, C.A.; Ito, M.; Ogata, T.; Ferguson-Smith, A.C. Genomic imprinting at the mammalian Dlk1-Dio3 domain. *Trends Genet*. **2008**, *24*, 306–316
2. Zeng, T.B.; He, H.J.; Han, Z.B.; Zhang, F.W.; Huang, Z.J.; Liu, Q.; Cui, W.; Wu, Q. DNA methylation dynamics of a maternally methylated DMR in the mouse Dlk1-Dio3 domain. *FEBS Lett.* **2014**, *588*, 4665–4671.

Disclaimer/Publisher's Note: The statements, opinions and data contained in all publications are solely those of the individual author(s) and contributor(s) and not of MDPI and/or the editor(s). MDPI and/or the editor(s) disclaim responsibility for any injury to people or property resulting from any ideas, methods, instructions or products referred to in the content.
